# Bradykinin-Mediated Angioedema: An Update of the Genetic Causes and the Impact of Genomics

**DOI:** 10.3389/fgene.2019.00900

**Published:** 2019-09-27

**Authors:** Itahisa Marcelino-Rodriguez, Ariel Callero, Alejandro Mendoza-Alvarez, Eva Perez-Rodriguez, Javier Barrios-Recio, Jose C. Garcia-Robaina, Carlos Flores

**Affiliations:** ^1^Research Unit, Hospital Universitario Nuestra Señora de Candelaria, Universidad de La Laguna, Santa Cruz de Tenerife, Spain; ^2^Allergy Unit, Hospital Universitario Nuestra Señora de Candelaria, Universidad de La Laguna, Santa Cruz de Tenerife, Spain; ^3^Instituto Tecnológico y de Energías Renovables (ITER), Genomics Division, Santa Cruz de Tenerife, Spain; ^4^CIBER de Enfermedades Respiratorias, Instituto de Salud Carlos III, Madrid, Spain; ^5^Instituto de Tecnologías Biomédicas (ITB), Universidad de La Laguna, Santa Cruz de Tenerife, Spain

**Keywords:** angioedema, inheritance, diagnosis, sequencing, precision medicine

## Abstract

Recurrent episodes of bradykinin-mediated angioedema (Bk-AE) can associate with acquired or hereditary conditions, the former most commonly developing secondarily to a pharmacological treatment. Despite successful genomic advances that have led to the identification of a large number of disease genes irrespective of disease prevalence, their application to Bk-AE has barely occurred. As a consequence, the genetic causes of Bk-AE remain poorly understood, obstructing the identification of patient subtypes and the development of precision medicine strategies. This review provides an update of the genetic studies completed to date on the acquired forms, which have almost exclusively focused on Bk-AE secondarily to the angiotensin-converting enzyme inhibitor treatment, and the blooming subdivision of the hereditary forms established by the identification of novel causal genes with next-generation sequencing (NGS) technology-based exome studies in genetically undiagnosed patients. Finally, based on the diverse benefits that are offered by the technology, we present arguments favoring the use of holistic NGS approaches as first-tier genetic tests as a promise to reduce the diagnostic odyssey of patients with suspected hereditary forms of Bk-AE.

## Introduction

**Figure 1:**
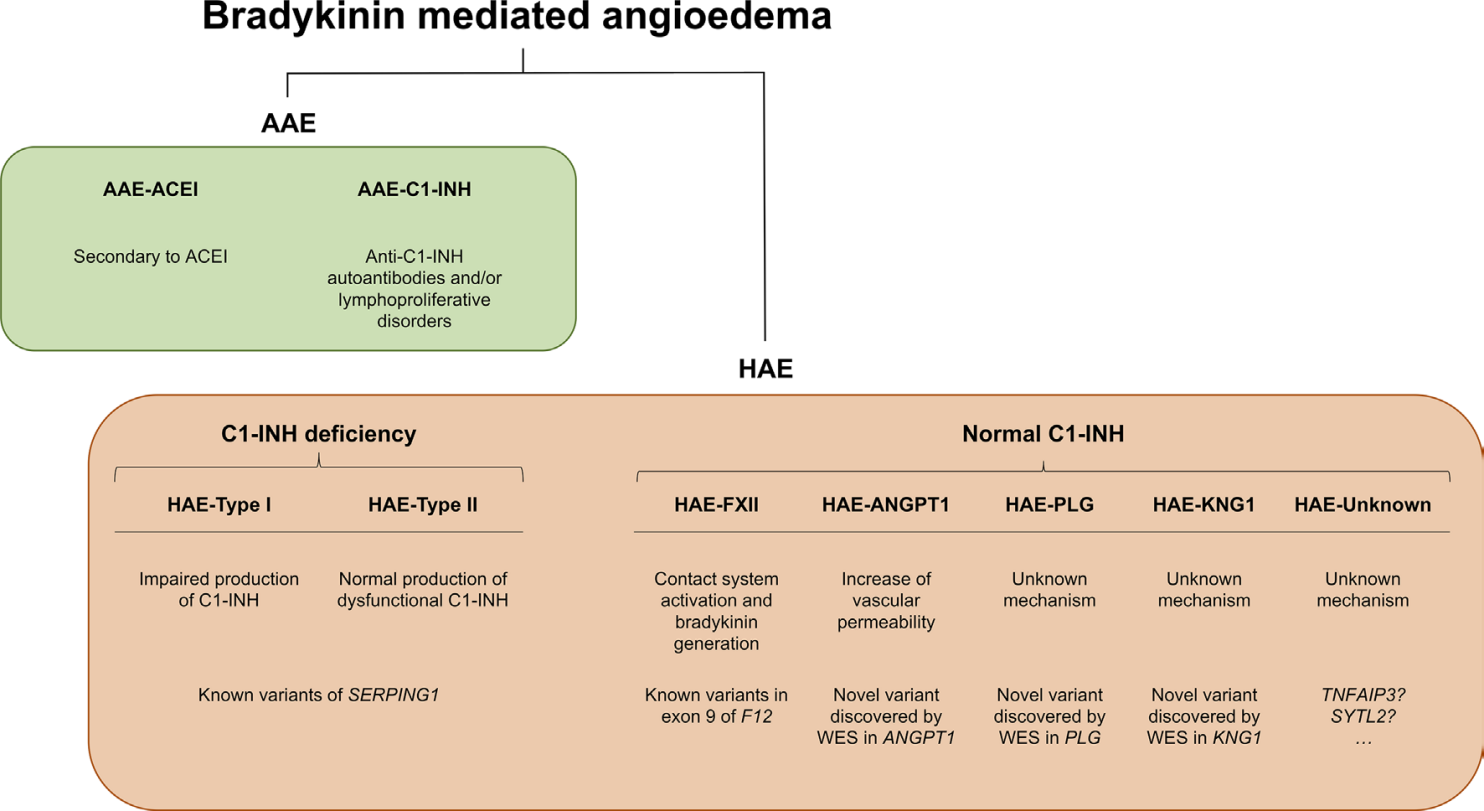
Schematic representation of bradykinin-mediated angioedema (AE) subtypes subdivided into acquired (AAE) and hereditary (HAE) forms. Two major groups of patients can be recognized within AAE forms based on the presence of a C1 inhibitor (C1-INH) deficiency or on a provocation by ACE inhibitors. Among the HAE forms, a major subdivision is made based on the existence of a C1-INH deficiency (caused by variants affecting function of *SERPING1*) or a normal C1-INH activity. The latter can be caused by diverse genes, most of them identified in recent whole-exome sequencing (WES) analyses of families. There remains a proportion of patients with unknown genetic causes where newly reported genes might explain disease causality. *SERPING1*, C1-inhibitor; *ANGPT1*, angiopoietin 1; *PLG*, plasminogen; *KNG1*, kininogen 1; *TNFAIP3*, tumor necrosis factor alpha-induced protein 3; *SYTL2*, synaptotagmin like 2.

Angioedema (AE) is most commonly defined as a self-limiting and localized edema of the subcutaneous and submucosal tissue, subsequent to blood vessel dilation and increased vascular permeability induced by vasoactive mediators. Approximately 30% of AE episodes are elicited by mast-cell mediators such as histamine (the so-called allergic pathway) (Zingale et al., 2006; Mansi et al., 2015). However, AE can be also mediated by bradykinin (Bk), a central mediator in the kinin–kallikrein system (i.e., the “nonallergic” pathway). Bk is a potent vasodilator that increases vascular permeability and mediates pain (Maurer et al., 2011; Magerl et al., 2014). It triggers the dilatation of blood vessels via the release of the endothelium-derived hyperpolarizing factor, prostacyclin, and nitric oxide. Therefore, the dysregulation of Bk homeostasis results in AE. Bk-mediated AE (Bk-AE), which is associated with the manifestation of recurrent episodes (Cicardi and Johnston, 2012; Cicardi et al., 2014), can be found in acquired (AAE) and hereditary (HAE) forms (**Figure 1**).

This review focuses on the genetics of Bk-AE in four main blocks: (1) main symptoms to clinically contextualize the topic; (2) the AAE forms and an overview of the current knowledge of the genetic factors involved in their susceptibility; (3) the causal genes of HAE forms and the recent knowledge improvements due to the use of next-generation sequencing (NGS) technologies; and (4) a discussion of the potential use of holistic NGS approaches as first-tier genetic tests for the diagnosis of hereditary forms.

## Main Symptoms

Bk-AE has been reported in all ethnic groups, usually affecting the skin, the gastrointestinal tract, or the upper airways. Bk-AE develops as a cutaneous swelling in almost three-fourths of the patients and as severe abdominal attacks in approximately one-fourth of them (Nzeako et al., 2001; Agostoni et al., 2004). Abdominal attacks can be very debilitating as they usually cause dramatic abdominal spasms (Frank et al., 1976; Savino et al., 2017). Attacks can even represent a life-threatening condition if the upper respiratory tract is affected by the edema, where it may cause obstruction of airways (Caballero et al., 2011a). The upper-airway episodes are the least frequent. However, they are the primary cause of mortality in Bk-AE patients. The AE episodes have different levels of frequency and severity and may develop spontaneously or may be triggered by stress, infections, trauma, medical or surgical interventions, or hormonal factors (pregnancy, menstruation, or the use of oral contraceptives). In 39% of the cases, patients admitted that the first episode developed after an identifiable traumatic event (Frank et al., 1976).

Among patients with HAE forms, the symptoms can develop during the infancy, although they are more frequently evidenced during the puberty. Approximately 50% of the patients will have the first episode at the age of 10–15 years (Caballero and Cabañas, 2016).

If AE patients are untreated, the attacks can be frequent, developing in a range from once a week to once a year and where each attack would typically last for a few days until it resolves spontaneously. As such, Bk-AE affects the quality of life. Overall, the estimates suggest that a Bk-AE patient can be debilitated by the symptoms between 20 and 100 days a year. Therefore, Bk-AE also associates with an important economic burden (Cicardi and Agostino, 1996).

## Acquired Forms of AE

One of the most common causes of Bk-AE is linked to a reduction of Bk degradation subsequent to the use of particular medications. This is the case of AAE forms due to the use of angiotensin-converting enzyme inhibitors (ACEIs), which are used for the treatment of cardiovascular conditions. This type of Bk-AE is usually referred to as AAE-ACEI. The angiotensin-converting enzyme (ACE) is critical in the degradation of Bk, which is hypothesized to accumulate excessively in some patients taking ACEI. In this case, the symptoms can happen at any moment of the treatment, despite the possibility that the patient may have been tolerating ACEI for months or years. The symptoms may develop within minutes or hours, and they usually last for 24–72 h. However, most patients show AE episodes within the first month after starting the ACEI treatment (Do et al., 2018). These patients usually have recurrent attacks with intervals that are free of symptoms between them. If the ACEI treatment is not withdrawn, the attacks will be more frequent and severe. Since its first description in 1984 (Jett, 1984), most AAE cases are diagnosed secondary to ACEI because of their wide prescription for treating hypertension, diabetes, ischemic cardiopathy, chronic kidney diseases, and systolic heart failure. Therefore, it is not surprising to find AAE episodes declared in clinical trials, meta-analysis, and observational studies (Mann et al., 2008; Makani et al., 2012; Banerji et al., 2017; Do et al., 2018). The prevalence of AAE has been estimated in 0.1–0.7% of patients taking ACEI. However, due to massive prescription of these drugs, up to 40% of the AE-related emergency department visits in the USA could be caused by the use of ACEI (Banerji et al., 2008). Although still with an unknown mechanism, there is evidence that other drugs can also trigger AE. This is the case for aliskiren (a renin inhibitor), which associates with AE in 0.4% of patients (White et al., 2010). This is also the case for dipeptidyl peptidase (DDP)-4 inhibitors or gliptins (sitagliptin, linagliptin, and saxagliptin) that are prescribed to patients with type 2 diabetes. Moreover, their effects are additive given that the risk of developing AE is larger in patients taking a DDP-4 inhibitor in combination with an ACEI (Brown et al., 2009).

Alternatively, an uncontrolled generation of Bk can also underlie AE pathogenesis. This is the case of the HAE forms and of the AAE forms that are due to a deficiency in the C1 inhibitor (C1-INH) (Craig et al., 2012). The C1-INH protein is a key protease inhibitor in the complement system that is also prominently involved in the regulation of vascular permeability. In AAE-C1-INH cases, the synthesis of the C1-INH protein is normal, albeit its catabolism is increased. AAE-C1-INH has a very low prevalence (around 1:500,000), and the cases have been associated with the existence of other diseases such as non-Hodgkin lymphoma or other B-lymphocyte-related abnormalities, which may lead to the production of neutralizing C1-INH autoantibodies and monoclonal gammopathies of undetermined significance (Zingale et al., 2006; Cicardi et al., 2014; Zanichelli et al., 2017).

## Genetics and the Acquired Forms of AE

The interplay between genetic factors and the environment (drug reactions, hormones, age, etc.) shapes Bk-AE susceptibility. As such, Bk-AE is a complex condition. As part of its genetic component, rare variants of a few genes associate with large effects in the phenotype (e.g., *SERPING1* mutations). This is the case of HAE forms that will be the subject of a more detailed discussion later on in this review. However, the genetic component of Bk-AE is also likely to be explained by a number of frequent genetic variants associated with weak effects in the clinical condition. However, while it is likely that frequent genetic variants act as modifiers influencing the clinical symptoms of HAE patients, frequent genetic variants are expected to constitute the main factors contributing to genetic susceptibility to the AAE forms, simply because of the AAE prevalence. While precise prevalence estimates of Bk-AE subtypes are difficult to obtain, a study among patients suspected to be Bk-AE from a French reference center (Dessart et al., 2015) indicated that approximately 74% of those patients could be related to AAE forms, given that they had no known variants affecting the function of HAE genes (screen limited to those known at the time) and had had no family history of AE. The reasoning underlying the idea has its origin in the common disease–common variant hypothesis (Manolio et al., 2009), which essentially supports that the genetic component of prevalent diseases can be explained by the combined influence of frequent variants with mild effects in the phenotype in hundreds or thousands of genes.

A PubMed search from 1970 until October of 2018 of the terms “angioedema” AND “genetics” or the terms “angioedema” AND “mutation” allowed us to retrieve 1,465 and 308 results, respectively. To focus on the Bk-AAE forms, we filtered out those publications that were reviews, clinical studies, studies in HAE cases, studies focusing on non-Bk-AE cases, or studies that were written in a language other than English. This left us with as few as 12 genetic studies of AAE (**Table 1**). Strikingly, most of them focused on AAE secondary to ACEIs. In the absence of high-resolution maps of genetic variation and without a possibility to access the current high-throughput genotyping or sequencing technologies, Duan et al. (2005) followed the classical linkage analysis method. They supported that a regulatory variant of the *XPNPEP2* gene was determinant for the plasma aminopeptidase P levels and that the same variant was a risk factor for AE. The rest of the publications retrieved described association studies, meta-analysis of results from published association studies, or case reports. However, all the studies have focused on particular variants from a few biological candidates (*ACE*, *BDKRB2*, *F12*, *F5*, and *XPNPEP2*), involving very narrow designs with limited sample sizes (average of 80 cases per study; not counting the meta-analysis and case reports) and lacking replication studies. The sole exception to the latter corresponds to a genome-wide association study (GWAS) published in 2013 (Pare et al., 2013) where the authors assessed hundreds of thousands of variants across the genome in a multiethnic study of 175 AAE-ACEI and 489 controls and a replication study conducted in a small sample of 19 AAE-ACEI cases and 57 matched controls. Results were negative, although a focused analysis in the genes encoding the Bk-degrading or substance P-degrading enzymes suggested a possible effect of variants in the gene encoding neprilysin (*MME*).

**Table 1:** Genetic studies of acquired bradykinin-mediated angioedema (Bk-AE) published until 2018.

Year	Type of study	Sample size (cases : controls)		Population	Gene(s)	Reference
		Discovery	Replication			
2017	Candidate gene in a case report	–	–	Multiethnic	*F12*	(Veronez et al., 2017)
2013	GWAS	175:489	19:57	Multiethnic	*MME* (top)	(Pare et al., 2013)
2013	Candidate gene	52:77	–	Multiethnic	*BDKRB2*	(Moholisa et al., 2013)
2013	Candidate gene^‡^	223:584	–	Multiethnic	*XPNPEP2*	(Mahmoudpour et al., 2013)
2011	Candidate gene	34:127	–	Multiethnic	*XPNPEP2*	(Cilia La Corte et al., 2011)
2010	Candidate gene	169:397	–	Multiethnic	*XPNPEP2*	(Woodard-Grice et al., 2010)
2010	Candidate gene	65:65	–	Unreported	*ACE, BDKRB2*	(Bas et al., 2010)
2008	Candidate gene	32:96	–	Unreported	*ACE*	(Gulec et al., 2008)
2008	Candidate gene	95:161	–	Multiethnic	*ACE*	(Akcali et al., 2008)
2006	Candidate gene in a case report	–	–	Unreported	*F5*	(Osmanaˇgaoˇglu et al., 2006)
2006	Candidate gene in families	14	–	Unreported	*XPNPEP2*	(Molinaro et al., 2006)
2005	Candidate gene^†^	20:60	–	European	*XPNPEP2*	(Duan et al., 2005)

GWAS, Genome-wide association study.

^†^Association study following a linkage analysis of a quantitative trait in families affected by Bk-AE.

^‡^Meta-analysis.

Taken together and because of the limited sample size assessed in most of these studies and the narrow genetic insight of all but one of them, the genetics of the AAE forms remains largely elusive. As such, genetic studies of AAE lack clinical utility at the moment. Such studies are conducted only for research purposes.

## Genetics of HAE

HAE was described for the first time in 1843 by Robert Graves. It follows a simple Mendelian inheritance [Online Mendelian Inheritance in Man (OMIM): 106100 and 610618]. Despite that, HAE still remains a largely unknown disease for the medical community, which has a negative impact on the precise and timely diagnosis and treatment of patients. One main cause of this lack of knowledge is its low prevalence, which has been estimated to be 1 in 50,000 on average and ranging from 1:10,000 to 1:150,000 (Nzeako et al., 2001; Gompels et al., 2005; Dreskin and Patel, 2014). Therefore, it is considered a rare disease (ORPHA: 91378) with an autosomal dominant inheritance, with variable penetrance, and where most of patients have abnormal C1-INH levels in plasma due to the presence of a variant of *SERPING1* gene affecting its function. This hereditary subtype is most commonly known as HAE type I and constitutes the classic presentation of the hereditary forms (**Figure 1**). Roughly 80% of HAE patients are still diagnosed by having a C1-INH deficiency. Another possible consequence of *SERPING1* variants affecting gene function has been described. While for HAE type I, these variants cause a quantitative decrease of the C1-INH levels in plasma, for HAE-type II, they cause a decrease in the activity of the protein (functional deficit) (**Figure 1**). In this case, C1-INH plasma levels may not be affected or can be elevated. This is because of a decrease in C1-INH catabolism that can cause an increase of its half-life (Cicardi et al., 2014; Caballero et al., 2015).

**Figure 2:**
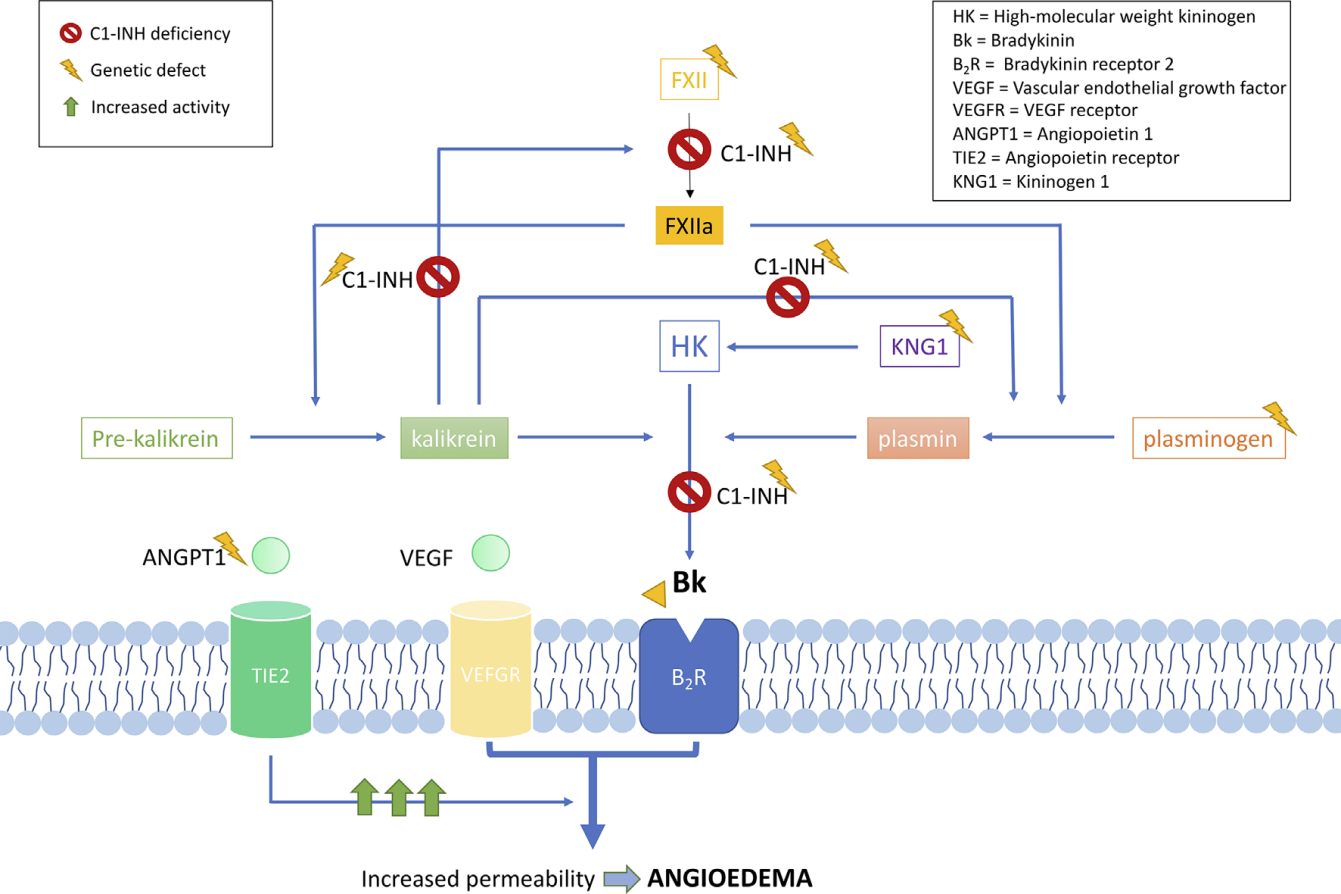
Schematic representation of the complement and contact system with simplified interactions of the protein activities encoded by the known hereditary angioedema (HAE) genes. Overall, all gene deficiencies lead to an increase in activity of bradykinin, which is a potent vasodilator that leads to an increase of vascular permeability and, therefore, to the formation of edema. C1 inhibitor (C1-INH) is a protease inhibitor with regulatory functions on the complement system and fibrinolysis. It is a key negative regulatory protein of the contact system, and the encoding gene is usually affected in HAE patients. HAE can be caused also by defects in the *F12* gene, causing a FXII proenzyme autoactivation and leading to an increase in all the mediators of the cascade. Recent exome studies have identified other causal genes of HAE, whose encoded products interact in the system, some of which are as follows: angiopoietin 1, where variants affecting function would reduce its binding capability to the receptor, leading to an enhanced vascular permeability by a variety of mediators [including vascular endothelial growth factor (VEGF)], rather than just by bradykinin; plasminogen, where variants translate into an enhanced binding to activators, triggering the fibrinolytic system with subsequent formation of bradykinin; and kininogen 1, where variants affecting function alter the generation of high-molecular-weight kininogen, increasing the half-life of bradykinin.

C1-INH is a glycoprotein that belongs to the superfamily of protease inhibitors (SERPINs) with regulatory functions on the complement system and the fibrinolysis. C1-INH is a key regulatory protein of the contact system (**Figure 2**). It inhibits different molecules such as the activated factor XII (FXIIa), kallikrein, and the activated factor XI (FXIa) (Ratnoff and Lepow, 1957; Schapira et al., 1982; de Agostini et al., 1984; Harpel et al., 1985). These others regulate the proteolytic cascades of the complement, the fibrinolysis, and the contact system by organizing inhibitory complexes of different proteases (C1r, C1s, and MASP1 in the complement system; FXIIa and kallikrein in the contact system; FXIa and thrombin in the coagulation; and plasmin and plasminogen activator in the fibrinolysis). C1-INH exerts its inhibitory function through the formation of equimolar irreversible complexes with the target proteases. It is able to dissociate the C1qC1r2–C1s2 complex (Sim et al., 1979). It also leads to a covalent complex C1-INH–protease, generating a conformational change that irreversibly traps the protease (Cicardi and Johnston, 2012). Because of these irreversible links, the presence of variants affecting the function of *SERPING1* in heterozygous patients leads to a 5–30% reduction of C1-INH activity compared to unaffected individuals, causing an uncontrolled activation of FXII. This, in turn, generates an excess of Bk levels, corresponding with a haploinsufficiency (Caballero et al., 2011a). It is anticipated that gene variants altering the activity of other proteins in these pathways might also be plausible biological causes of HAE.

**Figure 3:**
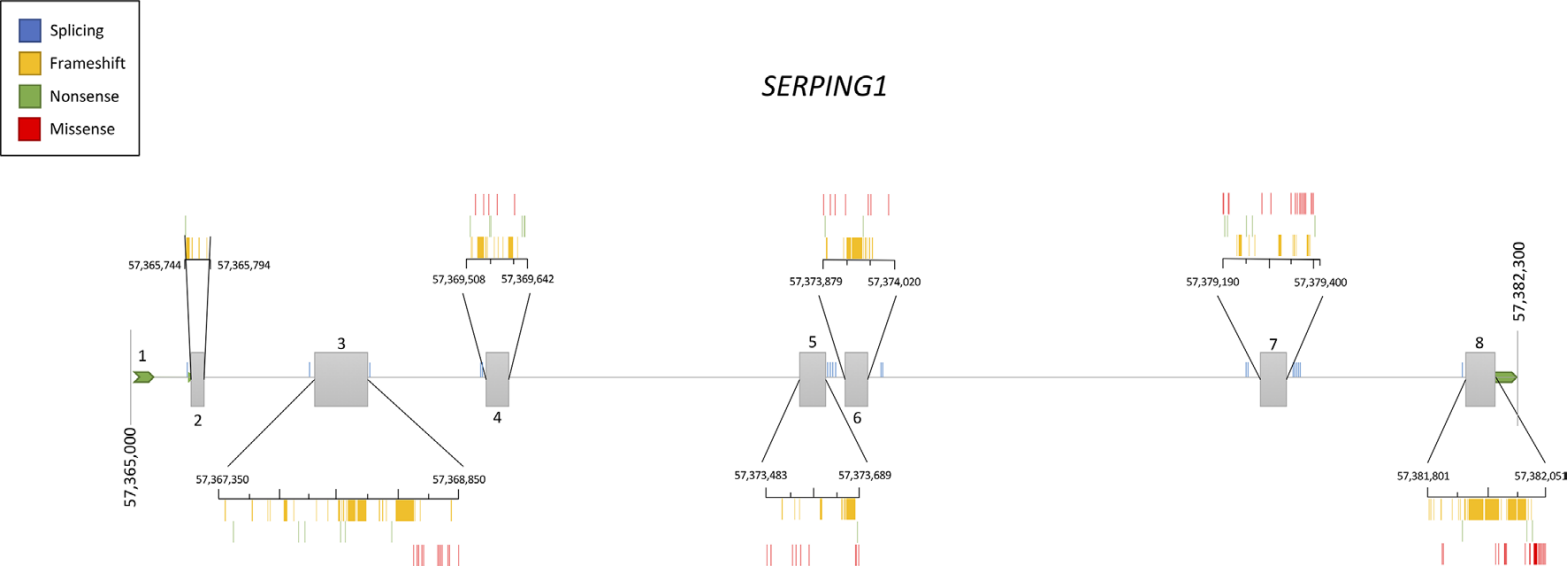
Schematic representation of the location of the pathogenic and likely pathogenic variants described so far in the *SERPING1* gene, encoding the C1 inhibitor, among patients with hereditary angioedema. Pathogenicity was inferred according to the American College of Medical Genetics and Genomics (ACMG) classification. Represented vertical bars correspond to the variants, which have been categorized by the effect (frameshift, nonsense, and missense). Splicing variants are also represented by vertical bars outside the exons. Exons are represented by gray boxes with the exon number indicated. Untranslated regions from 5′ and 3′ are also represented as an extended horizontal green arrow.

Genetic studies in hundreds of families have contributed to define the current knowledge of the *SERPING1* mutational spectrum causing HAE. This knowledge facilitates the identification of the variants affecting gene function in other patients with a suspected C1-INH deficiency, therefore contributing to improve the diagnosis of HAE. Based on the information provided by VarSome (Kopanos et al., 2019) and HAEdb (Kalmár et al., 2005) (accessed 09/10/2018), the variants affecting *SERPING1* function—classified as likely pathogenic or pathogenic according to the guidelines of the American College of Medical Genetics and Genomics (ACMG) (Richards et al., 2015)—are largely missense (40%) and frameshift (35%) changes, while as few as 9% predict splicing or nonsense changes (**Figure 3**). These proportions should be considered with caution given the difficulties in predicting pathogenicity of splicing variants (Shaikh et al., 2018). Most commonly, the variants affecting *SERPING1* function are single base substitutions. The exceptions are those causing frameshifts, which are due to insertions or deletions (indels) in the size range of 1–11 base pairs (bp). HAEdb also has records for 45 structural variants (SVs; 87% of them being deletions) affecting *SERPING1* that have caused HAE type I and HAE type II. Besides these, there is evidence of three deletions and one insertion that have affected the gene entirely. Remarkably, 62% and 20% of the SVs involve exons 4 and 8, respectively. Exon 8 also accumulates the largest proportion of single base substitutions and indels causing HAE according to HAEdb. Interestingly, most of the exon 8 variants are found in patients with HAE type II (Caballero and Cabañas, 2016).

The original description of roughly 20% of patients with a family history of Bk-AE but that showed active and normal plasma levels of C1-INH supported that there must be other causal genes of HAE (Binkley and Davis, 2000; Bork et al., 2000). Years later, a novel variant affecting function was described in exon 9 of the *F12* gene, encoding the coagulation factor FXII, in some families affected by Bk-AE but without a C1-INH deficiency (HAE-nC1-INH; also known as HAE type III in the past) (Dewald and Bork, 2006). After that, the role of *F12* in the activation of the contact system and Bk generation has gained relevance in HAE (Björkqvist et al., 2015; de Maat et al., 2016; Piñero-Saavedra et al., 2016; Veronez et al., 2018). A new subtype of HAE is now defined (HAE-FXII) (**Figure 1**), although only four variants affecting function have been described thus far in the gene: two different missense changes of codon p.Thr328, a deletion of 72 bp, and an 18-bp duplication (Dewald and Bork, 2006; Bork et al., 2011; Kiss et al., 2013; Bork et al., 2014). Remarkably, they all affect exon 9 of *F12*, which strongly supports that the encoded proline-rich domain of the protein is key in the pathogenesis of HAE. The most common variant affecting function is p.Thr328Lys, which causes a gain of function in the protein without altering plasma levels of C1-INH. This explains the rise of Bk levels in HAE patients with normal C1-INH levels (Cichon et al., 2006). It is noteworthy that at least 90% of HAE-nC1-INH cases are women and the exacerbation episodes are strikingly dependent on additional exogenous estrogens (Binkley and Davis, 2000), and there are cases with variants affecting the *F12* function that are estrogen dependent (Prieto-García et al., 2017). In fact, it is well known that elevated levels of FXII can enhance Bk formation once an exacerbation event is triggered, and estrogens have been related with the levels of FXII in blood (Gordon et al., 1980; Hoem et al., 1991; Citarella et al., 1996). Given this, it is expected that other subtypes of HAE also have higher phenotypic expression in women.

According to the current guidelines, genetic testing is only offered to HAE type II and HAE-nC1-INH patients. Around one-fourth of the HAE-nC1-INH families will carry a variant affecting *F12* function (**Figure 1**), while the genetic cause will be unknown in more than 70% of the families (HAE-unknown) (Bork et al., 2015).

## NGS to Fully Define HAE Genetics

**Figure 4:**
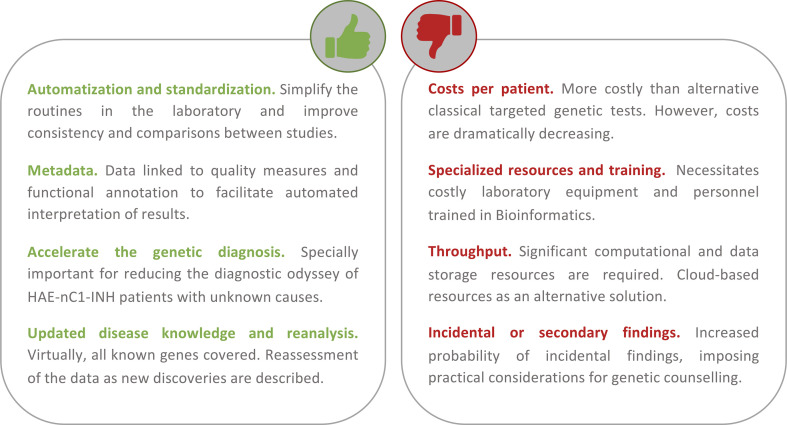
Summary of the main advantages and disadvantages of using a first-tier holistic next-generation sequencing (NGS) approach for the diagnosis of hereditary angioedema (HAE). Among the advantages, this approach reduces manual intervention (and the possibility to introduce errors during sample processing) and improves standardization. The routine analyses link the detection to diverse quality metrics of the results and rich biological information. These help to facilitate interpretations and reduce the possibility of reporting false positives. Because it is an unbiased assay (without a focus on genes or types of variation), it has the potential to identify novel HAE genes. If novel genes or novel variants affecting function are described, these can be re-analyzed in preexisting results from previous studies. If personalized medicine approaches are pursued, the results also permit the evaluation of other traits such as pharmacogenetics and inflammatory responses. Among the disadvantages, the cost of the test per patient continues to be higher than that of the classical genetic screens. But these costs are not considering the persons/hour costs nor the health system burden because of the genetic odyssey to reach a precise diagnosis. NGS requires specialized and costly laboratory and computational equipment and trained personnel to process the large amount of data generated. However, cloud-based solutions are proliferating, and they can drastically reduce this burden. There is an active debate around the additional burden of genetic counseling linked to the incidental findings that are expected.

The current marketed or custom-designed genetic tests used to screen for the genetic causes of HAE focus only on some or all of the exons of *SERPING1* and *F12* genes. These tests still rely mostly on the classic Sanger sequencing method for the identification of single base substitutions and indels, and multiplex ligation-dependent probe amplification (MLPA)-based or PCR-based techniques for the identification of alterations involving a larger sequence (for a list of available genetic diagnostic tests, see www.orphanet.net). However, since the introduction of NGS for high-throughput DNA sequencing, the decrease in the costs of sequencing and the analytical time required to identify the causal variants of a disease have transformed research and genetic diagnosis (Green et al., 2017; Stark et al., 2019). As a consequence, the genetic diagnosis of inherited diseases based on NGS tests is nowadays widespread in the clinic. The main reason for that is that NGS methods allow the simultaneous scanning of variants from as few as a dozen genes (referred to as gene panels) up to the entire genome. Sequencing of whole genomes would be seen by some as the less biased alternative. However, its use as a regular genetic test is still prohibitively expensive, besides entailing other challenges that are beyond the scope of this review. An alternative solution between these two extremes is whole-exome sequencing (WES) (Rabbani et al., 2014). WES is one of the most prolific applications of NGS as it allows a scanning of virtually all the exons from all known human genes (1–2% of the human genome). Some companies have also released a so-called clinical exome, a compact version of WES that targets the exons of approximately 4,000 OMIM genes. As WES focuses on screening the disease-related interpretable regions, it provides an efficient and affordable solution. It allows a simultaneous testing of all disease genes, and therefore, the results would not necessarily be limited to the genes that are known to cause the disease at the moment of testing (**Figure 4**). Thus, WES has the potential to identify new genetic causes of the disease and circumvents the problems arising from the lack of homogeneous gene tests (Hoang et al., 2018; Hosseini et al., 2018). It also provides broader results for additional analysis to be made once new knowledge of variants and disease genes is available. Besides these, WES data will be key to identifying unknown genetic modifiers of variable disease expressivity (**Figure 4**).

As a proof of concept, the recent application of WES to families that were classified as HAE-nC1-INH with unknown genetic causes revealed new causal genes. Bafunno et al. (2018) used WES for the first time to identify a novel variant affecting function in the angiopoietin 1 gene (*ANGPT1*, p.Ala119Ser) in an Italian family. This variant was detected in all affected members of the index family. However, it was very rare in the Exome Aggregation Consortium (ExAC) database. Of note, ExAC and the Genome Aggregation Database (gnomAD) constitute large-scale genome databases that assist in filtering out or flagging variants that are unlikely to be causal based on frequency criteria. As a matter of fact, only 3.4% and 1.3% of the variants affecting the function of HAE genes are listed in gnomAD and ExAC, respectively. The altered angiopoietin 1 formed less multimers and had a reduced binding capability to its receptor. In addition, a decreased ANGPT1/ANGPT2 ratio was found in patients carrying the p.Ala119Ser variant compared to unaffected controls. A plausible mechanism is that the vasculature of these patients would show an enhanced vascular permeability to a variety of mediators, rather than just to Bk. In a separate study, Cagini et al. (2018) also described Brazilian families with HAE that were carriers of two other rare *ANGPT1* variants with a pathogenic prediction (p.Ala8Val and p.Gln370His).

Another recent WES study in families with HAE-nC1-INH with unknown genetic causes identified the plasminogen gene (*PLG*) as a new causal gene (Bork et al., 2018). In this case, a p.Lys330Glu variant located in exon 9 was found in 14 German patients while it was absent from gnomAD. This variant predicted a change in the kringle 3 domain of plasminogen. The variant was found in all symptomatic patients and in nine out of 38 index patients from other independent families. In fact, two other studies identified the same variant in HAE cases from France and Japan (Belbézier et al., 2018; Yakushiji et al., 2018). Another study screened *PLG* for variants in eight unrelated index patients from Germany with HAE-nC1-INH with unknown genetic causes (Dewald, 2018). They also found the rare non-conservative missense variant in exon 9 (p.Lys311Glu) in three of the patients, using isoelectric focusing of plasma samples followed by an immunoblotting procedure, this study demonstrated that the presence of the p.Lys311Glu variant was associated with the presence of an aberrant plasminogen protein.

A recent study by Bork et al. (2019) described a WES study of two probands from a large family with HAE-nC1-INH with unknown genetic causes. They identified the hitherto unknown variant p.Met379Lys in exon 10 of the gene encoding kininogen 1 (*KNG1*), located in the cleave region for kinins such as Bk. The variant was present in all affected members, but it was absent in all the asymptomatic relatives; in other HAE-nC1-INH families that were carriers of variants affecting function in *F12*, *PLG*, or *ANGPT1* genes; and in gnomAD. The cosegregation analysis supported a dominant inheritance. Although the predictors of pathogenicity that were evaluated gave contradictory information, ACMG guidelines suggested it to be likely pathogenic.

Finally, Harris et al. (2018) also described a WES study in a Hispanic family with episodes of chronic urticaria and AE. Sequencing of the proband and of several family members revealed a variant in exon 2 of *TNFAIP3* (p.Arg22Gln) in all affected members but not in the unaffected mother. That variant was also extremely rare in gnomAD. Although the variant has an uncertain significance based on the ACMG guidelines (Richards et al., 2015), the gene encodes a mediator that is known to affect immune and inflammatory responses signaled by cytokines. In addition, previous studies have reported a statistical association of common variants of the gene with autoimmune diseases, allergy, asthma, and periodic fever syndromes.

Given this, it can be anticipated that new HAE genes are yet to be discovered as more laboratories adapt their standards to use holistic NGS methods to diagnose HAE. Taken as an example and although the evidence is still uncertain, the public resource of disease variants ClinVar (https://www.ncbi.nlm.nih.gov/clinvar/32061106) just reported a new variant affecting function in *SYTL2* gene (p.Ser297Thrfs) linked to a HAE case. Collectively, the new HAE genes discovered by WES so far explain the genetic cause in less than 5% of the diseased families. However, none of these newly identified genes have been incorporated yet to the guidelines for the routine diagnosis of HAE patients. Therefore, more robust estimates of their incidence are yet to be provided by the ongoing studies. In addition, further family studies and functionality assessments will be needed to firmly endorse a causality to these novel HAE genes. This will involve gene screens in other families (can be a part of WES studies) and cosegregation analyses in families. Additional biological knowledge from functional assays *in vitro* or *in vivo* in animal models may also be necessary.

## Is It Time for Genome Testing to Be at the First Line of HAE Diagnosis?

Typically, the patients with AE are treated in the emergency room as it would be a histaminergic AE attack. Therefore, AE patients are most commonly treated initially with corticosteroids, histamine blockers, and also with epinephrine if severe symptoms are present. None of these drugs are effective for HAE (Caballero et al., 2011b). Only if the patient does not respond to these first-line drugs are attenuated androgens (Danatrol®), plasma-derived C1-INH (Berinert®, Cinryze®, and Cetor®), or recombinant C1-INH (Rhucin® and Ruconest®) used. Other treatment options are authorized for the acute attacks and for prophylaxis, as the kallikrein inhibitor ecallantide (Kalbitor®) and Bk-2 receptor antagonist icatibant (Firazyr®). However, despite the published evidence demonstrating the benefits of using genetic tests, the first-tier diagnosis of HAE only involves plasma measurements separately in a period of 1–3 months of the C4 and C1-INH protein levels and of the C1-INH function (Caballero et al., 2011a). In fact, genetic tests are recommended for HAE diagnosis during the first months of life, as the C4 and C1-INH measurements are biased at that age (Caccia et al., 2014). For the patients with normal levels of C1-INH, it is also common to find that the diagnosis is purely based on clinical findings without the possibility of confirmatory laboratory tests. Precisely, in this subset of HAE patients is where the prolonged attacks predominate and where asphyxiation is more frequent (Craig et al., 2014). The observations also support that using genetic tests for HAE diagnosis also contribute to significantly reduce the time until an appropriate care is settled in the patients (Maia et al., 2017).

In the era of genomic medicine, we are witnessing a tremendous boost in the diagnosis of genetic diseases in terms of both the diagnostic yield and the significant reductions of the diagnostic odyssey. These, in turn, are contributing to a more efficient and precise management of patients. By leveraging the power of NGS-based methods in the clinic, genomic studies are allowing us to better predict outcomes and to obtain definitive diagnosis in many patients (Rabbani et al., 2014; van Dijk et al., 2014). In this context, there is a current debate of whether or not genomic tests should be considered as the first-line diagnostic tests (Stark et al., 2019) in substitution of other time-consuming, and sometimes invasive, procedures that require several hospital visits and a myriad of specialists. For the case of HAE, following the standard diagnosis guidelines usually results in an average diagnostic odyssey of 7 (USA) to 10–12 years (in France and Spain). The period is even longer (18 years) when the disease is caused by variants affecting a gene function other than the *SERPING1* (Caballero and Cabañas, 2016).

NGS-based screenings in general, and WES studies in particular, offer many benefits for gene discovery and for improving the diagnosis of patients with suspected HAE (**Figure 4**). Mendelian diseases with dominant inheritance, as is the case of HAE, impose a greater technical challenge for classic genetic analysis. This is because heterozygous variants are technically more difficult to detect and analyze. A recent analytical validation of an NGS-based carrier screen for cystic fibrosis demonstrated that NGS enabled accurate detection of the causal variants, achieving high sensitivity and specificity compared to the established genetic screening methods (Beauchamp et al., 2019). According to the experience of the International Rare Diseases Research Consortium (IRDiRC), the utility of WES for rare disease gene identification is beyond any doubt (Boycott et al., 2017). For many rare diseases that were previously intractable with classic approaches, WES studies have been able to evidence the genetic causes. This is extensible to HAE given that a few WES studies completed in the last 2 years have allowed to multiply by three the number of possible HAE genes. In this context, there are many approaches and tools that can be incorporated to HAE diagnosis to further assist in fully defining its genetic causes. One obvious one is to test the benefits of whole-genome sequencing, at least in particular situations, as they allow screening of genetic variations that remain obscure for WES studies. In fact, whole-genome sequencing and WES have been suggested as replacements for the classical first-line genetic tests in particular settings (Clark et al., 2018). The reason is the considerable cost savings, suggesting a cost of the test to be as few as 6% of the total costs of patient care (Splinter et al., 2018), compared to the alternative procedures (Goodwin et al., 2016; Green et al., 2017; Stark et al., 2019). Comparative studies as these are lacking in the literature for HAE patients. Another possibility is to leverage the human phenotype ontology (HPO; Köhler et al., 2017) and other platforms for genotype- and phenotype-driven matching algorithms, to facilitate the confirmatory studies in unrelated patients by permitting the remote match of cases with similar phenotypes and/or genetic variants.

Given the significant number of HAE patients that remain undiagnosed despite the genetic screenings using classic and narrow methods and the successful use of WES for revealing novel HAE genes, moving towards a first-tier genomics-based diagnosis of HAE is justified. Beyond that, genomics-based screenings have the potential to develop precision medicine strategies for HAE as they can also bridge the gap between genomic research and clinical care. For example, they would allow us to leverage the information from pharmacogenetics-associated alleles to estimate dose responses, which are particularly relevant in patients that may require multiple medications. Whether it will be desirable that a virtual panel of HAE genes extracted from the WES data is what should be analyzed at first instance before moving on to the complete genomic information obtained is a matter that will require further discussions. In terms of time and cost savings, holistic genomic solutions are also favored compared to classical targeted solutions since, while they both have similar opportunities to expose the different types of genetic variation, the persons/hour costs of laboratory procedures will be lower for the holistic solutions as there will be only one assay needed to cover the different genetic diseases that are assessed in a diagnostic setting.

## Conclusions

Bk-AE is a complex condition with a largely unknown genetic component. Despite a few genes explaining a large proportion of HAE cases, Bk-AE remains unfamiliar to the medical community. Establishing a definitive etiologic diagnosis in patients with suspected HAE is important for a timely implementation of therapies and for the development of precision medicine strategies. In many hospitals, genetic tests are not used on a regular basis for diagnosing HAE. When used, the tests are not used as the first tier and rely on narrow approaches that lack standards in the community. The major consequence is a long diagnostic odyssey for most patients and a heavy burden on the health-care system. As it has been recently demonstrated, the use of genetic tests that are based on holistic NGS methods has the potential to detect a much wider range of variants affecting the function of HAE genes. Updated recommendations will need to consider the pros and cons of applying holistic NGS approaches as a first-tier HAE diagnosis.

## Author Contributions

IM-R, AM-A, and CF revised the literature and the pathogenic potential of genetic variants. AC, EP-R, JB-R, J-GR, and IM-R contributed to the information for the clinical management and treatment of the patients. IM-R, AC, AM-A, and CF wrote the manuscript and designed the figures. All the authors revised and accepted the final version of the manuscript.

## Funding

This work was supported by the Ministerio de Ciencia, Innovación y Universidades (RTC-2017-6471-1; MINECO/AEI/FEDER, UE), which was co-financed by the European Regional Development Funds ‘A way of making Europe’ from the European Union, Area Tenerife 2030 from Cabildo de Tenerife (CGIEU0000219140), and by agreement OA17/008 with the Instituto Tecnológico y de Energías Renovables (ITER) to strengthen scientific and technological education, training, research, development, and innovation in genomics, personalized medicine, and biotechnology. AM-A was supported by a fellowship from ULL-Cajasiete. The content of this publication is solely the responsibility of the authors and does not necessarily reflect the views or policies of the funding agencies.

## Conflict of Interest

The authors declare that the research was conducted in the absence of any commercial or financial relationships that could be construed as a potential conflict of interest.
