# Corrigendum: Bradykinin-Mediated Angioedema: An Update of the Genetic Causes and the Impact of Genomics

**DOI:** 10.3389/fgene.2020.00690

**Published:** 2020-07-28

**Authors:** Itahisa Marcelino-Rodriguez, Ariel Callero, Alejandro Mendoza-Alvarez, Eva Perez-Rodriguez, Javier Barrios-Recio, Jose C. Garcia-Robaina, Carlos Flores

**Affiliations:** ^1^Research Unit, Hospital Universitario Nuestra Señora de Candelaria, Universidad de La Laguna, Santa Cruz de Tenerife, Spain; ^2^Allergy Unit, Hospital Universitario Nuestra Señora de Candelaria, Universidad de La Laguna, Santa Cruz de Tenerife, Spain; ^3^Instituto Tecnológico y de Energías Renovables (ITER), Genomics Division, Santa Cruz de Tenerife, Spain; ^4^CIBER de Enfermedades Respiratorias, Instituto de Salud Carlos III, Madrid, Spain; ^5^Instituto de Tecnologías Biomédicas (ITB), Universidad de La Laguna, Santa Cruz de Tenerife, Spain

**Keywords:** angioedema, inheritance, diagnosis, sequencing, precision medicine

In the original article, there was a mistake in Table 1 as published. The study conducted by Veronez et al. (2018) did not focus on acquired forms of angioedema (AAE). In addition, the study published in 2017 by the same author, describes a rare mutation detected within *F12* gene in a patient with angioedema induced by angiotensin-converting enzyme inhibitors. The reference (Veronez et al., 2018) has been modified to Veronez et al. (2017). Besides, one of the gene acronyms “*BDKRB2” was not set* in italics. This is has been corrected and shown in Table 1 below.

**Table 1:** Genetic studies of acquired bradykinin-mediated angioedema (Bk-AE) published until 2018.

		**Sample size (cases: controls)**				
**Year**	**Type of study**	**Discovery**	**Replication**	**Population**	**Gene(s)**	**References**
2017	Candidate gene in a case report	–	–	Multiethnic	*F12*	Veronez et al. (2017)
2013	GWAS	175: 489	19:57	Multiethnic	*MME* (top)	Pare et al. (2013)
2013	Candidate gene	52: 77	–	Multiethnic	*BDKRB2*	Moholisa et al. (2013)
2013	Candidate gene^‡^	223: 584	–	Multiethnic	*XPNPEP2*	Mahmoudpour et al. (2013)
2011	Candidate gene	34: 127	–	Multiethnic	*XPNPEP2*	Cilia La Corte et al. (2011)
2010	Candidate gene	169: 397	–	Multiethnic	*XPNPEP2*	Woodard-Grice et al. (2010)
2010	Candidate gene	65: 65	–	Unreported	*ACE, BDKRB2*	Bas et al. (2010)
2008	Candidate gene	32: 96	–	Unreported	*ACE*	Gulec et al. (2008)
2008	Candidate gene	95: 161	–	Multiethnic	*ACE*	Akcali et al. (2008)
2006	Candidate gene in a case report	–	–	Unreported	*F5*	Osmanagaoglu et al. (2006)
2006	Candidate gene in families	14	–	Unreported	*XPNPEP2*	Molinaro et al. (2006)
2005	Candidate gene^†^	20: 60	–	European	*XPNPEP2*	Duan et al. (2005)

† *Association study following a linkage analysis of a quantitative trait in families affected by Bk-AE. GWAS, Genome-wide association study*.

‡ *Meta-analysis*.

Also, we stated that in the study conducted by Dewald (2018) there is another rare variant affecting function detected within *PLG* gene. However, this is the same variant (p.Lys330Glu) described by Bork et al. (2018). This error was caused by the use of different nomenclature, where Bork et al., uses the correct nomenclature indicated by the Human Genome Variation Society guidelines. At the moment, only one PLG causal variant affecting function is reported in the scientific literature. A correction has been made to the third paragraph of Section: *NGS to Fully Define HAE Genetics*:

Another recent WES study in families with HAE-nC1-INH with unknown genetic causes identified the plasminogen gene (*PLG*) as a new causal gene (Bork et al., 2018). In this case, a p.Lys330Glu variant located in exon 9 was found in 14 German patients while it was absent from gnomAD. This variant predicted a change in the kringle 3 domain of plasminogen. The variant was found in all symptomatic patients and in nine out of 38 index patients from other independent families. In fact, two other studies identified the same variant in HAE cases from France and Japan (Belbézier et al., 2018; Yakushiji et al., 2018). Another study screened *PLG* for variants in eight unrelated index patients from Germany with HAE-nC1-INH with unknown genetic causes (Dewald, 2018). They also found the rare non-conservative missense variant in exon 9 (p.Lys330Glu) in three of the patients, using isoelectric focusing of plasma samples followed by an immunoblotting procedure, this study demonstrated that the presence of the p.Lys330Glu variant was associated with the presence of an aberrant plasminogen protein.

The authors apologize for this error and state that this does not change the scientific conclusions of the article in any way. The original article has been updated.
